# Supplementary material for: Complex Transposon Insertion as a Novel Cause of Pompe Disease
Source: Int J Mol Sci. 2021 Oct 8;22(19):10887. doi: 10.3390/ijms221910887 (PMC8509548; doi:10.3390/ijms221910887)
Supplement: Supplementary file 1 [file ijms-22-10887-s001.zip › Supplementary file.pdf]

**Amplification of the *GAA* cDNA:**
c1F-ATGAGGCAGCAGGTAGGACAG
c1R-AACTGGTCCGCAAAGAACAG
c2F-CACCGTCCCCACTCTACAG
c2R-TGGAACTCAGCCACCATGTC
c3F-GCCGCTGATTGGGAAGGTAT
c3R-GTCTGCAGGTCGTACCATGT
c4F-ACTCCTCCCCCACCTCTACA
c4R-ACTGCTCTCCCATCAACAGC

**qPCR for the *GAA* expression:**
exons 3-4:
q3F-CACCGTCCCCACTCTACAG
q3R-AACTGGTCCGCAAAGAACAG
exons 15-16:
q15F-ACTCCTCCCCCACCTCTACA
q16R-GGTCCACAGTCCAGGTGCTA

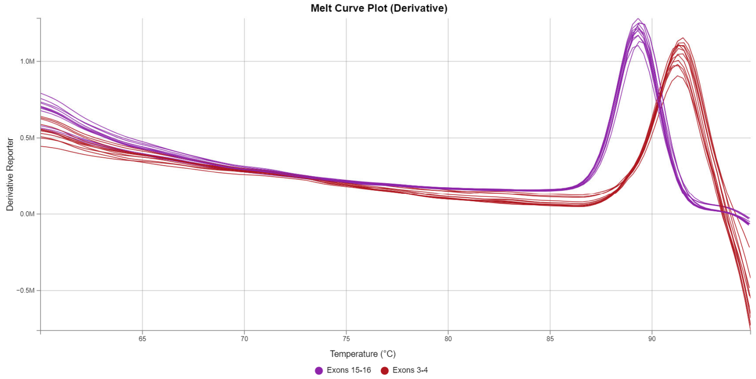


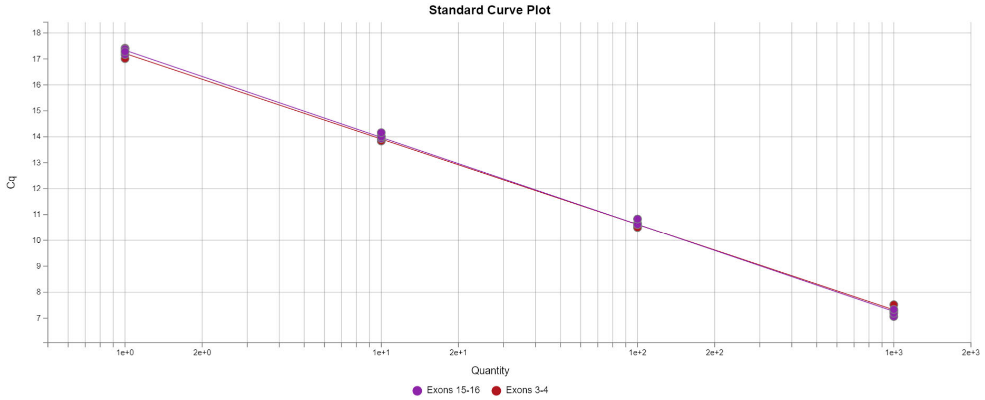


Target: Exons 3-4 Slop: -3.296 $R^2$: 0.999 Y-Inter: 17.209 Eff%: 101.103 Error: 0.034
Target: Exons 15-16 Slop: -3.357 $R^2$: 0.999 Y-Inter: 17.334 Eff%: 98.545 Error: 0.033

**qPCR for the chimeric mRNA isoform expression:**
GAA exons 14-15:
q14F-CGACGTCTGCGGCTTCCT
q15R-CCTGGGGCAGACTGAGCA
GAA exon 15-TE:
q15F-CTCTACACACTGTTCCACCAGG
TP5`R-TGAGGCAGGAGAATCAGGCA
TE 3`:
TP3`F-CCTCTCTAAACCAAGAGACCAC
TP3`R-GTTCAATTTGCCATTGTCCAGG

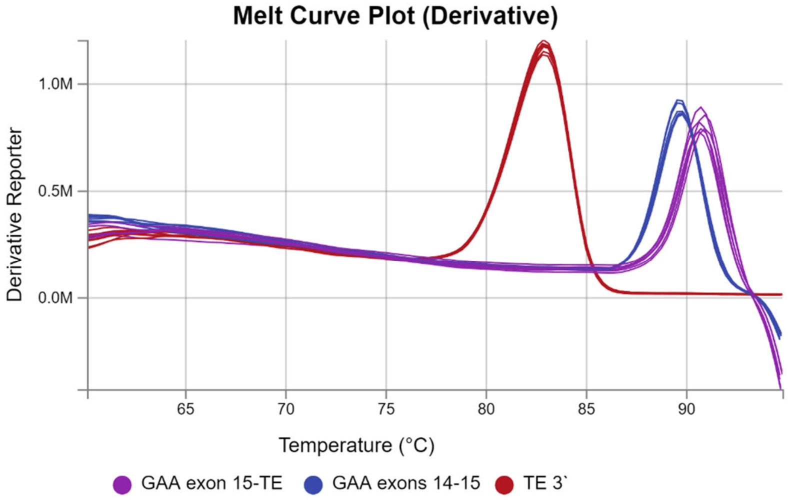


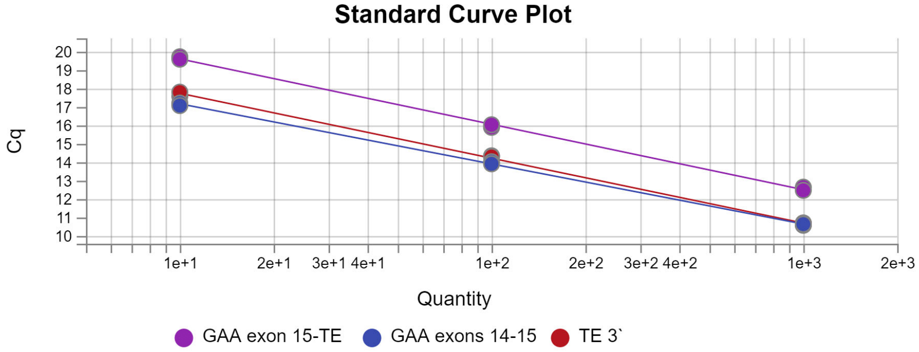


Target: TE 3` Slop: -3.524 $R^2$: 0.999 Y-Inter: 21.271 Eff%: 92.217 Error: 0.053

Target: GAA exon 15-TE Slop: -3.55 $R^2$: 0.999 Y-Inter: 23.164 Eff%: 91.296 Error: 0.062

Target: GAA exons 14-15 Slop: -3.263 $R^2$: 0.999 Y-Inter: 20.439 Eff%: 102.503 Error: 0.035

**qPCR for the *GAA* expression:**
EIF4A3q_F-TGGCCCAGACATCTGTAGAAA
EIF4A3q_R-AGAAAGAGCGGGAGTCCAT

**Bisulfite sequencing of GAA:**
exon 1:
bis1F-GATTTTTTAAATGTTGGTTGTTTT
bis1R-AACTAAAAATCTAAAACTCAAACTCC
bis1.2F-TTTTGGAATAATTGTGAGTTATGGAGTATA
bis1.2R-CCTCAACTTTCCCAACTAAAAAACC
exon 3-4:
bis3F-TTGGATTAGGATTATTTTGTGGAAT
bis4R-CTCCAATCTCCAAAACAAACAACAC
5` of intron 15:
bis15.1F-TGGTTTAGATAGAGGTAATTGTGTT
bis15.1R-ACCTACTAAATAAATAAAAAACCCC
3` of intron 15:
bis15.2F-TAGGAAATAGGATAGGGTAGAGTTG
bis15.2R-ATTCCAACAAATAAAAATCAATACC

**Gene specific primers for RACE:**

Race1F-CCTACTGGGAAGTGAGGAAAC
Race2F-TTACTGTGCAAGCTGGGATT
Race3F-CCTTTCAAGACCTAGTTTACTAAC

**Consensus sequence of the TE amplicon**
TCCCGTCTCCCTCTCCCTCTCCCGTCTCCCTCTCCCTCTCCCGTCTCCCTCTCCCTCTC
CCGNNNNNNNNNNCCTCTCCCTCTCCCTCTCCCTCTCCCTCTCCCTCTCCCTCTCCCT
CTCCCTCTCCGTCTCCGTCTCCGTCTCCCGTCTCCCTCCACGGTCTCCCTCTCAAGCC
GAGCCAAAGCTGGACGGTACTGCTGCCATCTCGGCTCACTGCAACCTCCCTGCCTGA
TTCTCCTGCCTCAGCCTGCCGAGTGCCTGCGATTGCAGGCACGCGCCGCCACGCCTG
ACTGGTTTTCGGTTTTTTTTTGGTGGAGATGGGGTTTCGCTGTGTTGGCCGGGCTGCT
CTCCAGCTCCTAACCGCGAGTGATCCGCCAGCCTCGGCCTCCCGAGGTGCCGGGATT
GCAGATGAAGTCTCGTTCACTCAGTGCTCAATGGTGCCCAGGCTGGAGTGCAGTGGC
GTGATCTCGGCTCNCTNCAANCTCCACCTCCCAGCCGCCTGCCTTGGCCTCCCAAAG
AGCCGAGATTGCAGCCTCTGCCCGGCCGCCACCCCGTCTGGGAAGTGAGGAGCGTC
TCTGCTTGGCCACCCATCGTCTGGGATATGAGGAGCCCCTCTGCCTGGCTGCCCAGT
GTGGAAAGTGAGGAGCGTCTCTGCCCGGCCGCCATCCCATCTAGGAAGCGAGAAGC
GCCTCTTCCCCGCCGCCATCCCATCTAGGAAGTGAGGAGCGTCTCTGCCCGGCCGCC
CATCGTCTGAGATGTGGGGAGCACCTCTGCCCCACCGCCCTGTCTGGGATGTGAGGA
GCGCCTCTGCTGGGCCGCAACCCTGTCTGGGAGGTGAGGAGTGTCTCTGCCCGGCCG
CTCCGTCTGAGAAGTGAGGAAACCCTCTGCCTGGCAACCGCCCCGTCTGAGAAGTG
AGGAGCCCCTCCGTCTGGCAACCACCCCGTCTGGGAAGTGAGGAGCGTCTCCGCCC
GGCAGCCACCCCGTCCGGGAGGGAGGTGGGGGGGGTCAGCCCCCCGCCCGGCCAGC
CGCCCCGTCCGGGAGGTGAGGGGCTCCTCTGCCCGGCCGCCCCTACTGGGAAGTGA
GGAGCCCCTCTGCCCGGCCAGCCGCCCCGTCCGGGAGGGAGGTGGGGGGGTCAGCC
CCCCGCCCGGCCGGCCGCCCCGTCCGGGAGGTGAGGGGCGCCTCTGCCCGGCCGCC
CCTACTGGGAAGTGAGGACCCCTCTGCCCGGCCAGTCGCCCCGTCCAGGAGGGAGG
TGGGGGGGTCAACCCCCCGCCCGGCCAGCCGCCCAGTCCGGGAGGGAGGTGGGGGG
TCAGCCCCCCGCCTGGCCAGCCGCCCCGTCCGGGAGGTGAGGGGCGCCTCTGCCCG
GCCGCCCCTACTGGGAAGTGAGGAGCCCCTCTGCCCGGCCAGCCGCCCCGCCCAGG
AGGGAGGTGGGGGGGTCAGCCCCCCGCCTGGCCAGCCGCCCCATCCGGGAGGGAGG
TGGGGGGGTCAGCCCCCCGCCCGGCCAGCCGCCNCGTCCGGGAGGGGGGAGGGGG
GGTCAGCCCCCTGCCCGGCCAGCCGCCCCGTCCGGGAGGGAGGTGGGGGGGGTCAG
CCCCCTGCCTGGCCAGCCGCCCCGTCCGGGAGGTGAGGGGCGCCTCTGCCCGGCCG
CCCCTACTGGGAAGTGAGGACCCCTCTGCCCGGCCAGCCGCCCCGTCCGGGAGGGA
GGTGGGGGGGGTCAGCACCCCGCCCGGCCAGCCGCCCCGTCCGGGAGGGAGGTGGG
GGGATCAGCCCCCTGCCTGGCCAGCCGCCCCGTCCGGGAGGTGAGGGGCGCCTCTG
CCCGGCCGCCCCTACTGGGAAGTGAGANACCCTCTGCCCGGCCAGCCGCCCCGTCC
GGGAGGGAGGTGGGGGGGGTCAGCACCCCGCCCGGCCAGCCGCCCCGTCCGGGAG
GGAGGTGGGGGGNTCAGCCCCCTGCCTGGCCAGCCGCCCCGTCCGGGAGGTGAGGG
GCGCCTCTGCCCGGCCGCCCCTACTGGGAAGTGAGGACCCCTCTGCCCGGCCAGCCG
CCCTGTCCGGGAGGGAGGTGGGGGGAACAGCCCCCCGCCCGGCCAGCCGCCCTATC
CAGGAGGTGAGGGGCGCCTCTGCCCGGCCGCCCCTACTGGGAAGTGAGGAGCCCCT
CTGCCTGGCCAGCCGCCCCGTCCGGGAGGGCGGTGGGGGGGTCAGCCCCCCGCCCG
GCCAGCCGCCCCATCTGGGAGGTGAGGGGCACTTCTGCCGGGCCGCCCCTACTGGG
AAGTGAGGAGCCCCTCTGCCCGGCCACGACCCCGTCTGGGAGGTGTGCCCAGCGGC
TCATTGGGGATGGGCCATGATGACAATGGCGGTTTTGTGGAATAGAAAGGCGGGAA
GGGTGGGGAAAAAATTGAGAAATCGGATGGTTGCTGGGTCTGTGTGGATAGAAGTA

GACATGGGAGACTTTTCATTTTGTTCTGTACTAAGAAAAATTCTTCTGCCTTGGGATC
CTGTTGATCTGTGACCTTATCCCCAACCCTGTGCTCTCTGAGACATGTGCTGTGTCCA
CTCAGGGTTAGATGGATTAAGGGCGGTGCAAGATGTGCTTTGTTGAACAGATGCTTG
AGGGCAGCATGCTCGTTGAGAGTCATCACCACTCCCTAATCTTAAGTACCCAGGGAC
ACAAACGCTGCGGAAGGCCGCAGGGTCCTCTGCCTAGGAAAACCAGAGACCTTTGT
TCACTTGTTTATCTGCTGACCTTCCCTCCACTATTGTCCTATGACCCTGCCAAATCCC
CCTCTGCGAGAAACACCCAAGAATGATCAATAAAAATAAAAAAAAAAAAAAAAAAA
AAAAAGAACTTGATTTAACATCTGTTGGTTCTGTCATATTTTCCAGGTTGCGTCTTCA
AATGTCTTGCCCCCAAAATACCAGTTTTTAAAATTAATACGTGATATTGTATCCTTGC
AAAATATTTGTAACATGTATGAAAGTTATGAACCTCTCTAAACCAAGAGACCACATC
AATCTAAAAGACAGATGTTATCAGGTTAGGGCCTCTTCCTGTGTGCTCCTCATCTTA
CTGTGCAAGCTGGGATTCCTGGACAATGGCAAATTGAACTGCTCTTCACAGATATCT
TTGACTTGATATTGATTTAAAATGAACTGCTTGCTTCTAAGTTTCCACCATTAAGAAT
TATGTACATTGTAGTAGATTTCTGGTAGTTAACTTTTATTCAGTTAAAGAAATTCCTT
TCAAGACCTAGTTTACTAACAATTTTGGTCAAGAATGGATGTTGAATTATAAAATTG
CTTTTTCTATGTCTATTATGAGAACTATTCTACTGTATTCTGTTTATTCAGTGAATTAC
ATTCTTGTTTCTTTTATTAAAAAAAAAAAAAAAAAAAAAAAAAAAAANAAAAAAAAA
AAAAAAAAAAGAAAAAANNNNNNNNNNNNNNNNNNNNNNNNNNNNNNNNNNNNN
NNNNNNNNNNNNNNNNNNNNNNNNNNNNNNNNAAAAAATAAAAAAAAAAAAAAAA
AAAAAAAA
