# Supplementary figures and images for: Complex Transposon Insertion as a Novel Cause of Pompe Disease
Source: Int J Mol Sci. 2021 Oct 8;22(19):10887. doi: 10.3390/ijms221910887 (PMC8509548; doi:10.3390/ijms221910887)

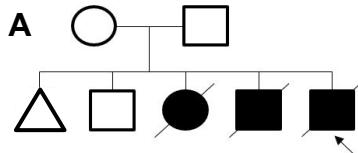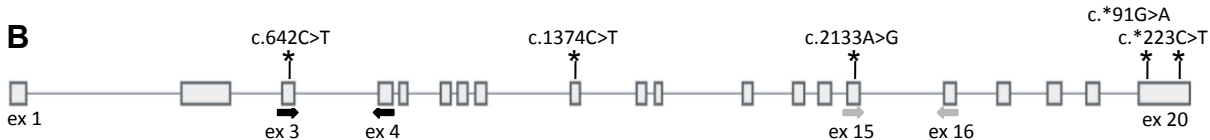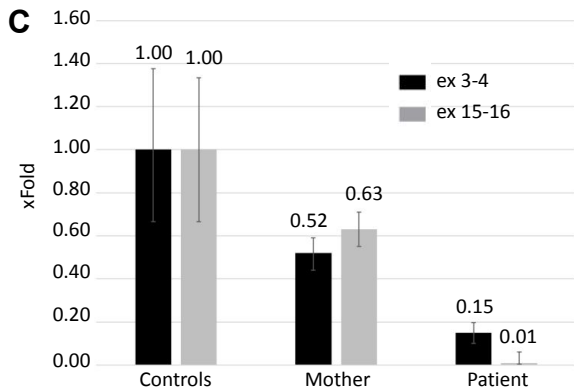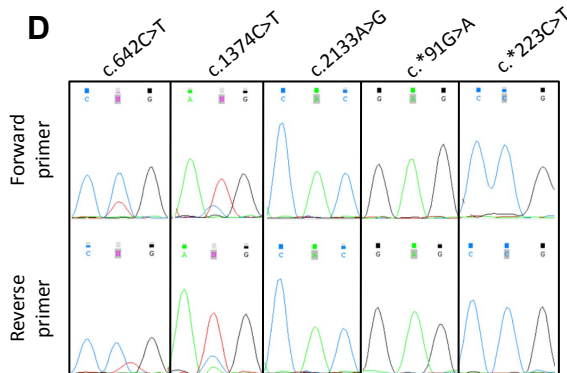

Supplement: Supplementary file 1 [file ijms-22-10887-s001.zip › Figure 1.pdf]

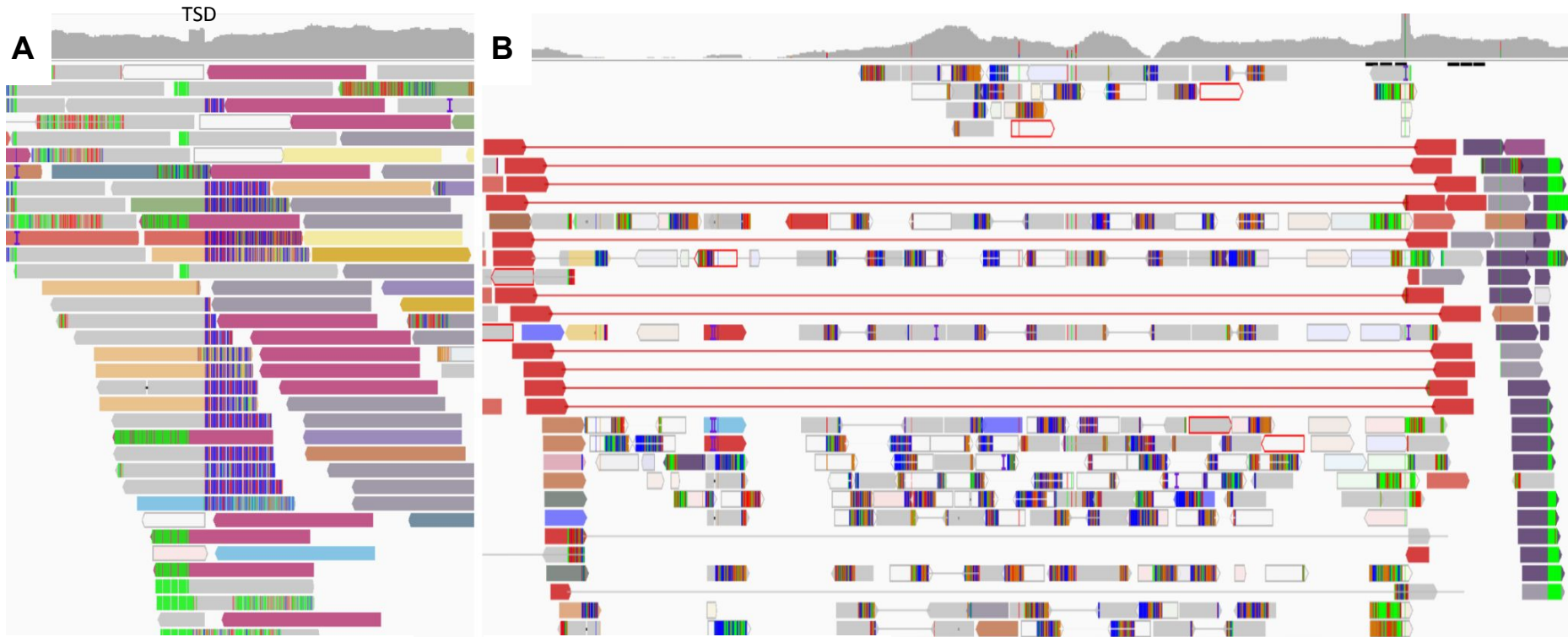

NC\_000017.11:80,114,000-80,114,447

NC\_000020.11:2,822,193-2,826,397

**C**

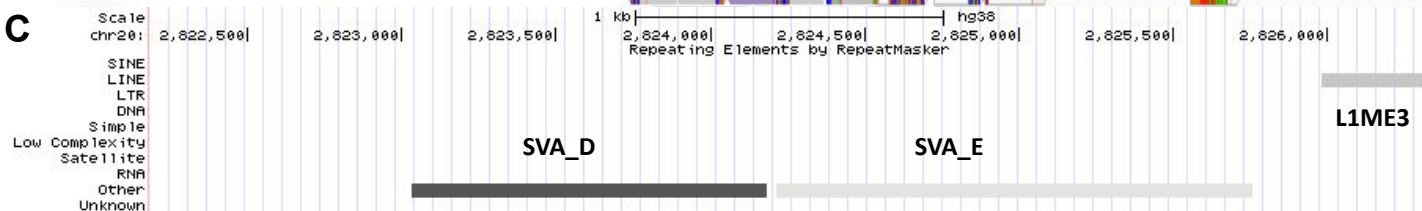

**D**

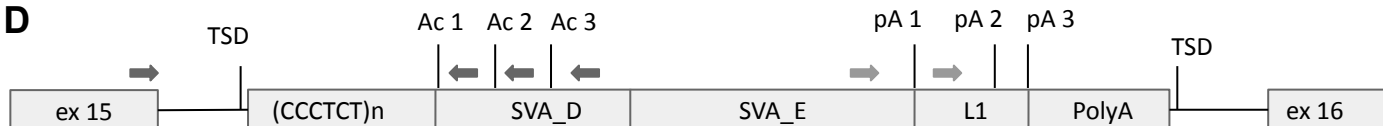

**E**

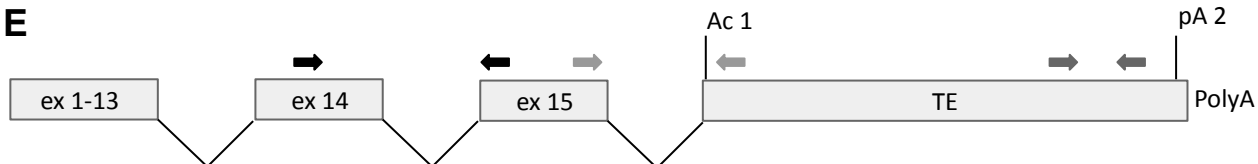

**F**

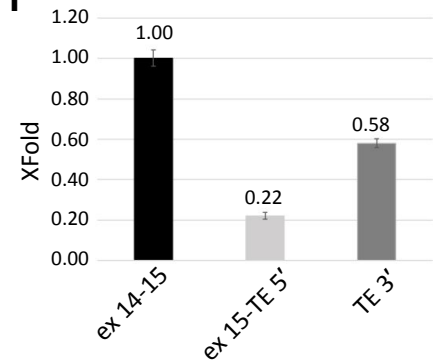

Supplement: Supplementary file 1 [file ijms-22-10887-s001.zip › Figure 2.pdf]
